# Crowdsourcing to support training for public health: A scoping review

**DOI:** 10.1371/journal.pgph.0002202

**Published:** 2023-07-26

**Authors:** Kadija M. Tahlil, Ucheoma Nwaozuru, Donaldson F. Conserve, Ujunwa F. Onyeama, Victor Ojo, Suzanne Day, Jason J. Ong, Weiming Tang, Nora E. Rosenberg, Titi Gbajabiamila, Susan Nkengasong, Chisom Obiezu-Umeh, David Oladele, Juliet Iwelunmor, Oliver Ezechi, Joseph D. Tucker

**Affiliations:** 1 Department of Epidemiology, University of North Carolina at Chapel Hill, Chapel Hill, North Carolina, United States of America; 2 Department of Implementation Science, Wake Forest University School of Medicine, Winston-Salem, North Carolina, United States of America; 3 Department of Prevention and Community Health, Milken Institute School of Public Health, The George Washington University, Washington, District of Columbia, United States of America; 4 Department of Health Behavior, University of North Carolina at Chapel Hill, Chapel Hill, North Carolina, United States of America; 5 Clinical Sciences Department, Nigerian Institute of Medical Research, Lagos, Nigeria; 6 Department of Medicine, Division of Infectious Diseases, University of North Carolina at Chapel Hill, Chapel Hill, North Carolina, United States of America; 7 Central Clinical School, Monash University, Melbourne, Australia; 8 Melbourne Sexual Health Centre, Alfred Health, Melbourne, Australia; 9 Department of Clinical Research, London School of Hygiene and Tropical Medicine, London, United Kingdom; 10 Institute of Global Health and Infectious Diseases, University of North Carolina at Chapel Hill, Chapel Hill, North Carolina, United States of America; 11 Department of Behavioral Science & Health Education, College for Public Health and Social Justice, Saint Louis University, St. Louis, Missouri, United States of America; 12 Clinical Research Department, Faculty of Infectious and Tropical Diseases, London School of Hygiene and Tropical Medicine, London, United Kingdom; The University of Sydney, AUSTRALIA

## Abstract

Crowdsourcing is an interactive process that has a group of individuals attempt to solve all or part of a problem and then share solutions with the public. Crowdsourcing is increasingly used to enhance training through developing learning materials and promoting mentorship. This scoping review aims to assess the literature on crowdsourcing for training in public health. We searched five medical and public health research databases using terms related to crowdsourcing and training. For this review, the concept of crowdsourcing included open calls, designathons, and other activities. We used a PRISMA checklist for scoping reviews. Each full-text was assessed by two independent reviewers. We identified 4,071 citations, and 74 studies were included in the scoping review. This included one study in a low-income country, 15 studies in middle-income countries, 35 studies in high-income countries, and 11 studies conducted in multiple countries of varying income levels (the country income level for 12 studies could not be ascertained). Nine studies used open calls, 35 used a hackathon, designathon or other “a-thon” event, and 30 used other crowdsourcing methods, such as citizen science programs and online creation platforms. In terms of crowdsourcing purpose, studies used crowdsourcing to educate participants (20 studies), develop learning materials (17 studies), enhance mentorship (13 studies) and identify trainees (9 studies). Fifteen studies used crowdsourcing for more than one training purpose. Thirty-four studies were done in-person, 31 were conducted virtually and nine used both meeting options for their crowdsourcing events. Seventeen studies generated open access materials. Our review found that crowdsourcing has been increasingly used to support public health training. This participatory approach can be a useful tool for training in a variety of settings and populations. Future research should investigate the impact of crowdsourcing on training outcomes.

## Introduction

In 2019, the World Health Organization (WHO) conducted a crowdsourcing open call to identify practical strategies to enhance research mentorship in low- and middle-income countries (LMICs) [1]. Open calls are an interactive form of crowdsourcing [2], which is a process that involves a group of individuals solving all or part of a problem, then sharing those solutions with the community [3]. The WHO open call solicited strategies to improve research mentorship and professional development, which were then assessed based on pre-specified criteria [1]. This open call received over 100 strategies, identified three individuals to contribute to a practical guide, engaged dozens of LMIC research institutions, and identified numerous open-access learning materials [1]. This underscores the ways that crowdsourcing approaches can enhance training and highlights the feasibility of crowdsourcing to enhance training engagement.

There is an increased recognition that we need to provide inclusive training to support diverse trainees [4]. There is a need to develop innovative approaches to identify early career investigators and nurture their opportunities for research [5], and to do so in participatory and inclusive ways [6]. Crowdsourcing approaches are one way to enhance training. Crowdsourcing has been previously used to identify LMIC researchers for training opportunities and engagement in health research as part of the WHO/TDR global programme [7, 8]. Crowdsourcing approaches have also been used in various other learning contexts, including developing learning materials [9], identifying open-access training resources [10], and identifying ways to enhance public health education and mentorship [1, 11, 12]. Training programs may benefit from crowdsourcing approaches that enhance community engagement, spur innovation, and identify learners. Traditional training programs often involve experts delivering one-way instruction and guidance to trainees with the aim of enhancing their personal and professional development. Crowdsourcing can be used to shift from conventional training approaches to a more open and collaborative process. Instead of experts being primarily responsible for training methods and outcomes, a diverse group of individuals from the community can work together to frame training strategies. Crowdsourcing to support training can help to prepare public health practitioners for interdisciplinary partnerships and provide access to community-developed resources.

Despite the growing interest in the potential of participatory approaches such as crowdsourcing in promoting training, few studies have examined the application of crowdsourcing for public health training purposes. There is little comprehensive understanding of the characteristic components of this approach for training, as well as its best practices, outputs, and outcomes. Although there are several empirical articles on the use of crowdsourcing approaches to promote public health training, no efforts have been made to collate and synthesize this body of knowledge. Amidst the growing importance of innovation in public health training, crowdsourcing approaches could potentially provide innovative and participatory training modalities and components. Hence, this scoping review investigates and summarizes the extent to which crowdsourcing has been used to support and promote public health training and explores critical components of how crowdsourcing can be used to improve public health training.

## Methods

### Search strategy

We organized a scoping review of the literature, drawing on the framework of Arksey and O’Malley [13] and following the PRISMA Extension for Scoping Reviews (S1 Checklist). We registered the protocol for the scoping review in the Open Science Framework (Registration DOI: https://doi.org/10.17605/OSF.IO/Q3PNH). A scoping approach was selected given substantial differences in the training methods and outcomes, several different ways of using crowdsourcing that preclude pooling, and many gaps in the literature. On March 14^th^, 2022, we conducted an initial search of five medical and public health research databases–PubMed, CINAHL, Embase, Global Health and Cochrane Library. We conducted a secondary search on April 5^th^, 2023 to capture any new articles published during the year after our initial search. The search algorithm included variations of the following terms: crowdsourcing, hackathon, designathon, training, education, mentorship, and capacity building (S1 Text). We identified and adapted these search terms from prior crowdsourcing and training review literature. Included publications focused on using crowdsourcing methods and training. We used the WHO/TDR definition of crowdsourcing: “the process of having a large group, including experts and non-experts, solve a problem and then share the solution with the public [14].” This definition is grounded in a broader crowdsourcing set of approaches [15]. This includes open calls (also known as innovation challenges or contests), designathons (also known as hackathons or sprint-like events), and other forms of crowdsourcing. For this paper, we define training broadly to encompass formal education, informal education, mentorship, coaching, and capacity-building for a wide range of ages and learning backgrounds [16]. We exported records from our search, removed duplicates using EndNote X9, and performed online screening.

### Study selection

Inclusion criteria were the following: relevant to public health; clear description of crowdsourcing methodology; the overall purpose was to enhance training, education, mentorship, or a related area. This included empirical descriptions of crowdsourcing training programs, clinical trials that used crowdsourcing methods for education or training, and descriptions of methods. There were no geographic or time restrictions on the search. Studies, commentaries/editorials, and opinion pieces that described potential programs that have not been implemented were excluded. We excluded systematic, scoping, or narrative reviews and studies that were not written in English.

Two reviewers independently reviewed titles and abstracts for inclusion, and a third reviewer was available to resolve discrepancies. Then two independent reviewers examined full-text manuscripts, excluding studies based on the criteria above. Data were extracted about the purpose of crowdsourcing and health topic of the crowdsourcing event. We also extracted information on the following components of crowdsourcing that can support public health training: crowdsourcing method (open call, designathon, other), country income level, type of crowdsourcing event (digital, in-person or both), and whether open access materials were generated. We categorized crowdsourcing based on the Joint International Consensus Statement on Crowdsourcing [17]. Subsequently, a narrative synthesis of the extracted data was performed. The stages of our narrative synthesis included: 1) descriptive statistics to summarize the extent and nature of included studies and 2) thematic categorization, which involved identifying common training areas and grouping studies based on those training categories.

## Results

### Identification of studies

**Fig 1:**
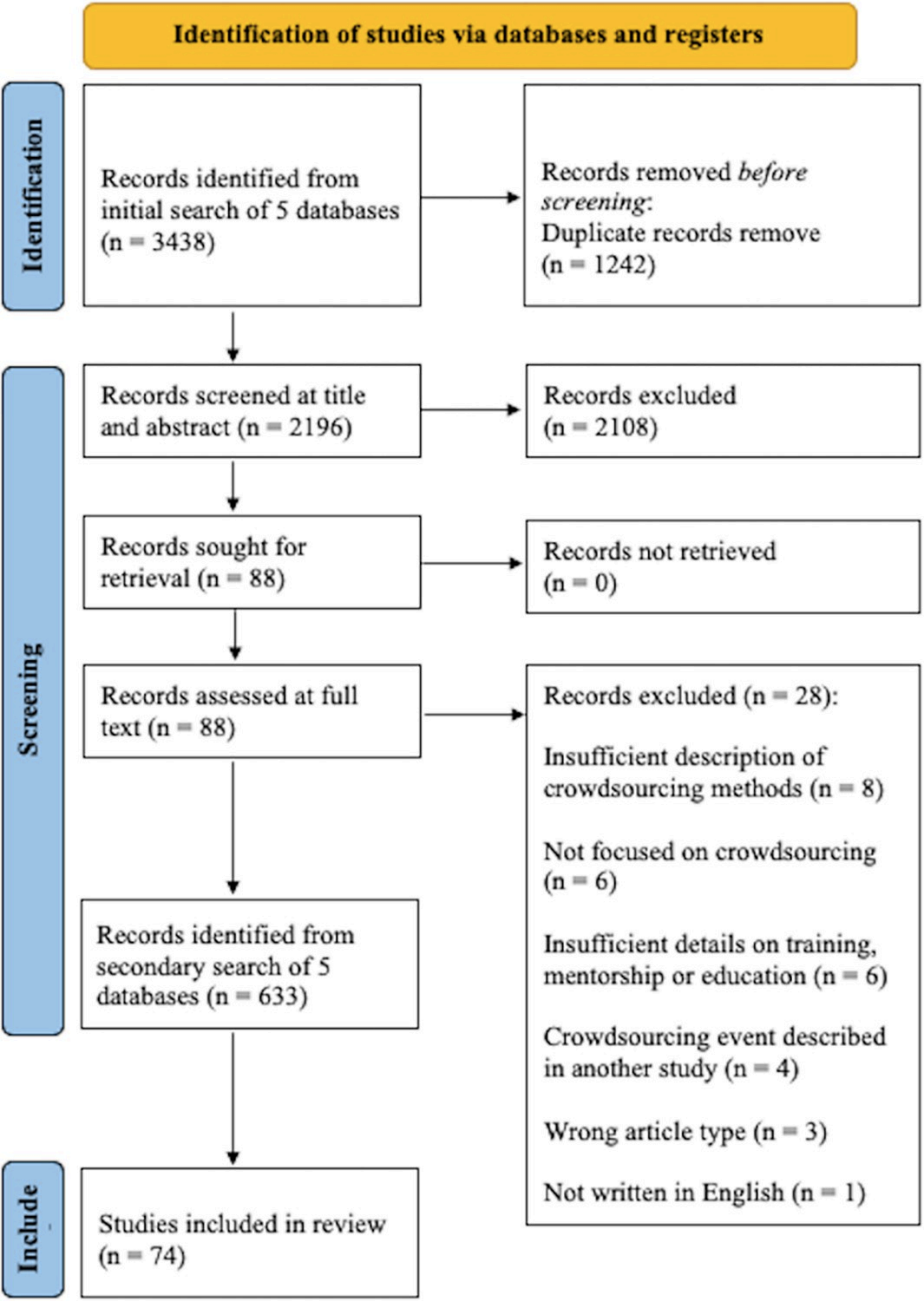
PRISMA flow diagram. The total number of records identified from the initial (n = 3,438) and secondary (n = 633) searches is 4,071. The secondary search was performed after records from the initial search were assessed at full text review.

We retrieved 3,438 publications from our initial database searches. After removing duplicates, the publications were screened for the relevance of the title and abstract, resulting in the exclusion of 2,108 publications. We further evaluated 88 publications for full-text eligibility. Of these, 28 articles were excluded for the following reasons: insufficient description of crowdsourcing methods (n = 8), not focused on crowdsourcing (n = 6), insufficient details on training, mentorship or education (n = 6), wrong article type (n = 3), not written in English (n = 1), and same crowdsourcing event already described in another study (n = 4). We retrieved 633 publications from our secondary database searches. The total number of publications retrieved from the initial and secondary database searches was 4,071. Overall, 74 studies were included in this review (Fig 1).

### Descriptive characteristics

Table 1 shows the descriptive characteristics of the 74 studies included in this review. The majority of studies were conducted in high-income countries (56.5%), followed by middle-income countries (24.2%). Eleven studies (17.7%) used crowdsourcing approaches in multiple countries of varying income levels and one study (1.6%) was conducted in a low-income country. Thirty-five studies (47.3%) used a hackathon or other “a-thon” event as the crowdsourcing method. Thirty studies (40.5%) used crowdsourcing methods other than open calls, hackathons, or other “a-thon” events. These other methods mainly included online creation platforms, citizen science programs, and peer groups. The majority of these crowdsourcing events took place in-person (45.9%). Only 17 studies (23%) identified in this review generated open access materials. The majority of studies (81%) were published in 2018 or after.

**Table 1:** Characteristics of studies using crowdsourcing for public health training between 2011–2023 (N = 74).

	n	%
**Country Income Classification**		
Low-income	1	1.6
Middle-income	15	24.2
High-income	35	56.5
Multiple income levels (Multiple countries)	11	17.7
Missing	12	
**Crowdsourcing Method**		
Hackathon/Designathon/Other "a-thon" events	35	47.3
Open call	9	12.2
Other	30	40.5
**Type of Crowdsourcing Event**		
In-person	34	45.9
Virtual	31	41.9
Both	9	12.2
**Purpose of Crowdsourcing**		
Education	20	27.0
Develop learning materials	17	23.0
Promote mentorship	13	17.6
Identify trainees	9	12.2
Multi-purpose	15	20.3
**Open Access Materials Generated**		
No	57	77.0
Yes	17	23.0

### Training areas

**Fig 2:**
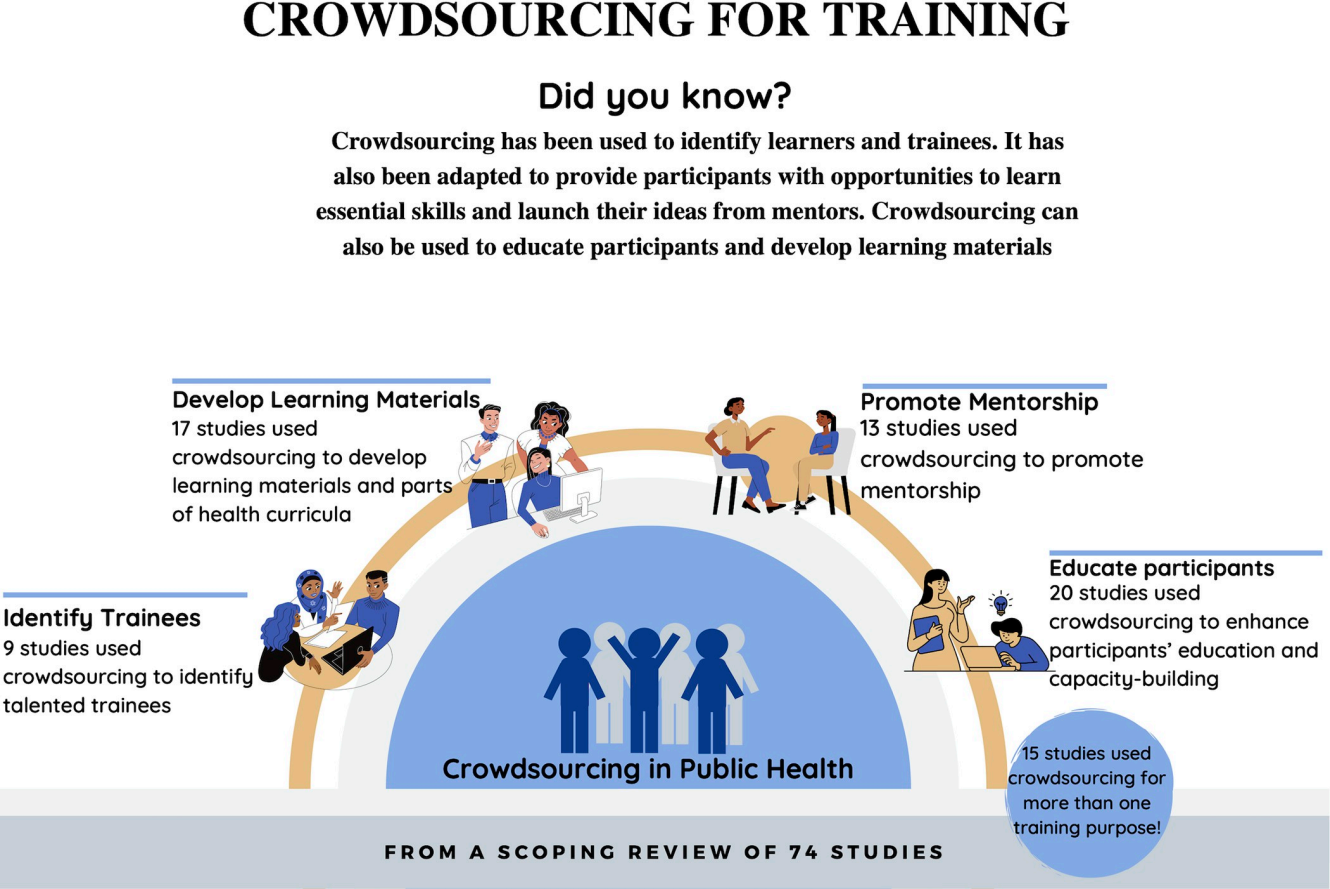
Crowdsourcing for public health training infographic.

We identified the use of crowdsourcing to support four areas of public health training (Fig 2). Crowdsourcing was used to 1) educate participants, 2) develop learning materials, 3) promote mentorship, or 4) identify trainees. Below, we describe the role of crowdsourcing for each of these training areas in further detail.

### Crowdsourcing to educate participants

Twenty studies (27%) used crowdsourcing to enhance participants’ education and capacity-building (Table 2) [18–37]. Of these studies, 12 were solely conducted in middle- or high-income countries. Twelve studies educated participants in-person, six were done virtually, and two used both methods. Eight studies used a hackathon or datathon to educate participants and five studies utilized citizen science programs. Hackathons were used to deliver a variety of educational content, including healthcare innovation [18], social work education [36], and neuroscience [20]. The Mount Sinai Health Hackathon, which is a 48-hour team-based competition that occurs annually, was a crowdsourcing event that serves as a model for team science education [23, 26]. This hackathon brought together individuals from different disciplines to work on a shared health problem, which fostered an environment of experiential learning through collaboration and communication. Also, three of the five studies that used citizen science programs to crowdsource for education were focused on environmental health. These studies educated high school students on topics such as air pollution [21], radiation monitoring [24], and radon exposure [28]. Upon completion of the educational portion of the programs, these students engaged in citizen science by going into their communities and collecting and reporting environmental data.

**Table 2:** Summary of studies that used crowdsourcing to educate participants (N = 20).

First Author	Publication Year	Country Income Level*	Number of Participants	Crowdsourcing Method	Type of Event	Open Access Materials Generated	Health Topic
Blindenbach-Driessen, F.	2014	Not specified	30	Hackathon	In-person	No	Healthcare innovation
Cai, H.	2022	MIC	172	Classroom curriculum	In-person	No	Global health
Craddock, RC.	2016	MIC/HIC	Not specified	Hackathon	In-person	Yes	Neuroscience
Ellenbug, JA.	2019	LIC/MIC/HIC	~11200	Citizen science program	Both	Yes	Air pollution
Estes, CF.	2021	Not specified	3817	Online creation platform	Virtual	Yes	Radiation oncology
Fattah, L.	2020	HIC	76	Hackathon	In-person	No	Rare diseases
Fojtíková, I.	2019	HIC	Over 30	Citizen science program	In-person	No	Radiation monitoring
Fuhrmeister, ER.	2021	HIC	Not specified	Classroom project	In-person	No	Antimicrobial resistance
Gabrilove, JL.	2018	HIC	87	Hackathon	In-person	No	Cancer
González, SA.	2022	MIC	97	Citizen science program	In-person	No	Health promotion
Hahn, EJ.	2020	HIC	27	Citizen science program	In-person	No	Radon exposure
Ischia, J.	2019	Not specified	Not specified	Online creation platform	Virtual	No	Cancer
Matthews, AK.	2022	HIC	8	Citizen science program	Virtual	No	Cancer
Piza, FMT.	2018	MIC	49	Datathon	In-person	No	Healthcare databases
Preiksaitis, C.	2023	HIC	12	Hackathon	In-person	No	Medical innovation
Puius, YA.	2023	HIC	Not specified	Work group	Virtual	Yes	Infectious diseases
See, C.	2014	Not specified	Not specified	Online and offline creation platforms	Both	No	Medical education
Sherbino, J.	2015	Not specified	86	Online journal club	Virtual	No	Medical education
Wilson, J.	2019	HIC	32	Hackathon	In-person	No	Homelessness
Zou, Y.	2022	LIC/MIC/HIC	Not specified	Hackathon	Virtual	No	Radiation oncology

*LIC = low-income country; MIC = middle-income country; HIC = high-income country

### Crowdsourcing to develop learning materials

Seventeen studies (23%) used crowdsourcing to develop learning materials and parts of health curricula (Table 3) [11, 38–53]. Of these studies, 11 were solely conducted in high-income countries. Fourteen studies only hosted the crowdsourcing event virtually. Ten studies made their learning materials publicly available. Nine studies utilized online creation platforms where participants collaborated to produce, review, collate and share learning materials. The use of online creation platforms resulted in the development of various learning resources, such as demonstration videos for learners on performing common pediatric procedures in resource-constrained settings [40], a cancer genetics e-textbook co-created by and for undergraduate students [49], a healthcare curriculum as part of a diagnostic radiography programme [50], and a mobile vascular surgery handbook that can help users access information during conferences and clinical care [52]. Also, one study that used crowdsourcing to develop learning materials was a global open call to solicit questions, infographics, and images to create open-access materials on antimicrobial resistance [11]. High-scoring entries from this open call were shared as learning resources with the public.

**Table 3:** Summary of studies that used crowdsourcing to develop learning materials (N = 17).

First Author	Publication Year	Country Income Level*	Number of Participants	Crowdsourcing Method	Type of Event	Open Access Materials Generated	Health Topic
Adsul, P.	2023	HIC	Not specified	Work group	Virtual	Yes	Health equity
Bate, A.	2017	MIC/HIC	Not specified	Online creation platform	Virtual	No	Pharmacovigilance education
Bensman, RS.	2017	HIC	Not specified	Online creation platform	Virtual	Yes	Global health
Berk, J.	2018	HIC	Not specified	Online creation platform	Virtual	Yes	Medical education
Drasovean, Y.	2021	Not specified	Over 2500	Open call	Virtual	No	Coronavirus disease 2019
Herodotou, C.	2018	HIC	Not specified	Online creation platform	Both	Yes	Citizen inquiry
Ianni, PA.	2020	HIC	Not specified	Online creation platform	Virtual	No	Translational science
Ilagan‐Ying, YC.	2022	HIC	67	Clinic education development	In-person	No	Medical education
Kercheval, JB.	2021	HIC	42	Curriculum development	Virtual	No	Medical education
Kpokiri, EE.	2021	MIC/HIC	Not specified	Open call	Virtual	Yes	Antimicrobial resistance
Leonard, HL.	2023	LIC/MIC/HIC	49	Hackathon	Virtual	No	Parkinson’s disease
Seam, N.	2019	HIC	Not specified	Online creation platform	Virtual	Yes	Medical education
Seid-Karbasi, P.	2017	HIC	89	Online creation platform	Both	Yes	Cancer genetics
St John-Matthews, J.	2020	HIC	27	Online creation platform	Virtual	No	Radiography
Staziaki, PV.	2022	Not specified	Not specified	Hackathon	Virtual	Yes	Radiology education
Sutzko, DC.	2019	HIC	54	Online creation platform	Virtual	Yes	Vascular surgery
Tangcharoensathien, V.	2020	Not specified	1483	Global online consultation	Virtual	Yes	Infodemics

*LIC = low-income country; MIC = middle-income country; HIC = high-income country

### Crowdsourcing to promote mentorship

Thirteen studies (17.6%) used crowdsourcing to promote mentorship (Table 4) [1, 8, 9, 13, 54–62]. Five of these studies were conducted in high income countries. Eight studies were conducted virtually, four were done in-person, and one used both methods to host their event. Eight studies used hackathons, three used open calls, and two used peer groups as their crowdsourcing method. All eight studies that organized hackathons provided mentors for their participants [8, 9, 54, 55, 58, 60–62]. The mentors had varying roles, which included providing expert knowledge, training participants in public speaking and presentation skills, reviewing ideas and prototypes, offering encouragement and support, and connecting participants to their networks. Two studies initiated open calls related to research mentorships; one solicited ideas to enhance research mentorship in LMICs [1] and another gathered data to understand and improve the impact of a global health research training program on trainees and students [13]. Peer groups were also vehicles to promote mentorship for internal medicine residents [56, 57]. Peer mentorships were used to promote professional development and support resident well-being.

**Table 4:** Summary of studies that used crowdsourcing to promote mentorship (N = 13).

First Author	Publication Year	Country Income Level*	Number of Participants	Crowdsourcing Method	Type of Event	Open Access Materials Generated	Health Topic
Bao, H.	2020	LIC/MIC/HIC	44	Open call	Virtual	No	Research mentorship
Bolton, WS.	2021	HIC	123	Hackathon	Virtual	Yes	Coronavirus disease 2019
Braune, K.	2021	HIC	48	Hackathon	Virtual	No	Coronavirus disease 2019
Buteau, A	2019	Not specified	Not specified	Peer group	In-person	No	Professional development
Ciccariello, C.	2018	Not specified	180	Peer group	Both	No	Well-being in residency
DePasse, JW.	2014	HIC	Not specified	Hackathon	In-person	No	Healthcare innovation
Goel, S.	2022	MIC	150+	Open call	Virtual	No	Hypertension
Koszalinski, RS.	2021	Not specified	1812	Hackathon	Virtual	No	Coronavirus disease 2019
Oppong, E.	2021	LIC/MIC	123	Open call	Virtual	No	Research mentorship
Poncette, AS.	2020	HIC	30	Hackathon	In-person	No	Healthcare innovation
Ruzgar, NM.	2020	HIC	31	Hackathon	In-person	No	Surgery
Tan, RKJ.	2022	MIC	Not specified	Designathon	Virtual	No	Global health
Ulitin, A.	2022	MIC	28	Hackathon	Virtual	No	Coronavirus disease 2019

*LIC = low-income country; MIC = middle-income country; HIC = high-income country

### Crowdsourcing to identify trainees

Nine studies (12.2%) used crowdsourcing methods to identify talented trainees for further opportunities, making training programs more inclusive (Table 5) [63–71]. Of these nine studies, three used open calls, four used hackathons, and two used citizen science programs to identify trainees. Five studies were conducted in-person, one was done virtually, and three used both methods to implement the crowdsourcing event. Five studies were solely conducted in high-income countries. Previous open calls that used crowdsourcing to identify trainees invited key stakeholders to offer their solutions on how to increase women’s participation in infectious diseases research fellowships [69], how to promote HIV self-testing among young people in Nigeria [70], how to improve the use of evidence-based practices in a behavioral health system [71]. In these past crowdsourcing open calls, participants with high-quality and promising ideas were invited to refine, finalize, present, and potentially implement their solutions through feedback and collaboration with content experts. These crowdsourcing methods were inclusive as they aggregated the ideas and perspectives of the trainees with the knowledge and experiences of the experts.

**Table 5:** Summary of studies that used crowdsourcing to identify trainees (N = 9).

First Author	Publication Year	Country Income Level*	Number of Participants	Crowdsourcing Method	Type of Event	Open Access Materials Generated	Health Topic
Amat, M.	2021	HIC	98	Hackathon	In-person	No	Healthcare challenges
Askins, N.	2020	HIC	Not specified	Citizen science program	Virtual	No	Cancer
Cooper, K.	2018	HIC	200	Hackathon	In-person	No	Radiology
Fadlelmola, FM.	2021	MIC	24	Hackathon	In-person	No	Genomic medicine and microbiome
Hidalgo-Ruz, V.	2013	HIC	983	Citizen science program	In-person	No	Marine environment research
Li, C.	2020	MIC	38	Hackathon	In-person	No	Health care utilization
Liu, E.	2020	LIC/MIC/HIC	Not specified	Open call	Both	No	Infectious diseases research
Rosenberg, NE.	2021	MIC	769	Open call	Both	No	Human immunodeficiency virus
Stewart, RE.	2019	HIC	55	Open call	Both	No	Evidence-based clinical practices

*LIC = low-income country; MIC = middle-income country; HIC = high-income country

### Crowdsourcing for more than one training purpose

Fifteen studies (20.3%) used crowdsourcing for more than one training purpose (Table 6) [72–86]. Of these studies, thirteen used hackathons or other “a-thon” events to achieve their training goals. Twelve studies were conducted in either a middle- or high-income country. All but three study’s crowdsourcing events were hosted in-person. The multiple training purposes in 13 of these studies included promoting research mentorship. One study used a crowdsourcing workshop in India to identify trainees and build their capacity to provide mental health services [76]. These trainees were identified after applying for the workshop and then paired with mentor experts who guided them through developing a mental health research funding proposal.

**Table 6:** Summary of studies that used crowdsourcing for more than one training purpose (N = 15).

First Author	Publication Year	Country Income Level*	Number of Participants	Training Purpose	Crowdsourcing Method	Type of Event	Open Access Materials Generated	Health Topic
Babatunde, A.	2023	LIC/MIC	44	Identify trainees; Promote mentorship	Hackathon	Virtual	No	Health equity
Butt, WA.	2020	LIC	116	Identify trainees; Promote mentorship	Hackathon	In-person	No	Medical education
Callisto, D.	2018	MIC	Not specified	Learning materials; Promote mentorship	Hackathon	In-person	No	Human immunodeficiency virus; viral hepatitis
Euzébio De Lima, C.	2016	MIC	Not specified	Education; Promote mentorship	Hackathon	In-person	No	Human immunodeficiency virus; Sexually transmitted diseases
Hawk, M.	2017	MIC	24	Identify trainees; Promote mentorship	Grantathon	In-person	No	Mental health
Jordan, RC.	2011	HIC	82	Identify trainees; Education	Citizen science program	In-person	No	Conservation science
Kahn, MJ.	2022	Not specified	37	Education; Promote mentorship	Open call	Virtual	No	Medical education
Lewis, S.	2021	MIC	90	Education; Promote mentorship	Hackathon	In-person	No	Cancer
Pathanasethpong, A.	2017	MIC	140	Education; Promote mentorship	Hackathon	In-person	Yes	Mobile health technology
Ramadi, KB.	2019	HIC	72	Identify trainees; Promote mentorship	Hackathon	In-person	No	Health diplomacy
Schwerdtle, P.	2018	HIC	300	Education; Learning materials	Mapathon	Both	Yes	Global health
Silver, JK.	2016	HIC	102	Education; Promote mentorship	Hackathon	In-person	No	Rehabilitation medicine innovation
Tahlil, KM.	2021	MIC	42	Identify Trainees; Promote mentorship	Designathon	In-person	No	Human immunodeficiency virus
Wang, JK.	2018	HIC	257	Education; Promote mentorship	Hackathon	In-person	No	Clinical needs
Wang, JK.	2018	MIC/HIC	245	Education; Promote mentorship	Hackathon	In-person	No	Medical innovation

*LIC = low-income country; MIC = middle-income country; HIC = high-income country

## Discussion

This scoping review describes the extent and characterizes existing research on crowdsourcing for public health training. We found that crowdsourcing has been used to support four areas of training: to educate participants, develop learning materials, promote mentorship, and identify trainees. Studies in this review featured different crowdsourcing approaches to improve public health training. We found that these participatory approaches have supported training on a broad range of health topics, including environmental health [21, 24, 28], infectious diseases [11, 25, 54, 55, 60, 69, 70, 74, 75, 84], and mental health [76]. This scoping review extends the literature on crowdsourcing to examine how it has been used to benefit public health training.

The findings of this scoping review provide an evidence base for the role of crowdsourcing to foster education and develop learning materials. These studies used a variety of crowdsourcing approaches such as hackathons, citizen science programs at schools, and online platforms to deliver education. These studies suggest that crowdsourcing can provide a structured method for community health and medical education. We also found that all studies that used crowdsourcing to develop learning materials used online collaboration systems. This demonstrates that crowdsourcing can be used to engage diverse online communities and provide a virtual environment where these communities can work together to develop training resources. Moreover, online crowdsourcing approaches may be less resource-intensive than in-person crowdsourcing events, which could be potentially useful in resource-limited settings. Crowdsourcing comes with an obligation to give back to the public who created the idea. Therefore, learning materials that were developed from crowdsourcing can then be used for future education and capacity-building. In the 13 studies that used crowdsourcing to develop learning materials, eight studies made their materials widely available to the public [11, 40, 41, 43, 48, 49, 52, 53], which allows the resources to be accessible without restrictions.

We also found that crowdsourcing has been used to promote research and professional mentorship. A recurring theme among studies focused on promoting mentorship is the use of participatory approaches to increase community participation in research and development of interventions. Crowdsourcing events have been used to identify participants with promising ideas and connect them with mentors who can help them iteratively refine their ideas. Typically, this would include events where participants practice pitching their ideas to these mentors and receive tailored feedback. Also, an additional advantage that can arise from these crowdsourcing events is the opportunity for mentees to have access to their mentors’ networks. This can help mentees strengthen their research ideas and broaden their own professional networks. It is also important for public health trainers to carefully consider and employ the most appropriate crowdsourcing method to enhance mentorship. Hackathons, for example, are a popular crowdsourcing approach to provide mentorship. Considerations for hackathons to successfully promote mentorship can include determining the appropriate mentor-mentee ratio for the event and incorporating designated and adequate mentoring sessions into the hackathon agenda.

Moreover, we found that crowdsourcing is a useful approach to identifying and engaging talented trainees. Open calls, in particular, can be used to select individuals for training. For example, one study used a crowdsourcing open call to solicit ideas on how to improve HIV self-testing among youth [70]. Participants with promising ideas were selected as finalists and proceeded to subsequent stages of the study for further development of their ideas and opportunities for apprenticeships. Another study used an open call to solicit ideas on improving the participation of women in an infectious disease research fellowship [69]. The top ideas from this open call were then implemented in the fellowship’s next application cycle, which saw an increase in the number of women applicants. Open calls have the potential to reach a wide and varied audience, which can provide an opportunity for enhanced inclusivity and engagement of early investigators. These methods should be considered by public health researchers as suitable approaches to identify and engage with early investigators.

Our scoping review had several limitations. First, the crowdsourcing literature is diverse in form and content, making the pooling of studies for meta-analysis difficult. Second, we captured fewer studies from low- and middle-income countries. This may be the result of fewer crowdsourcing activities in those countries or less reporting of those experiences. Third, our scoping review did not include grey literature, thus relevant studies may have been missed. Fourth, we may not have captured some capacity-building programs that used crowdsourcing but were not formally evaluated.

Our findings have implications for public health research, programming, and education. Crowdsourcing can serve as an innovative model to advance public health training. Crowdsourcing provides a way to go beyond conventional didactic approaches to training to engaging and collaborative methods to training. This may result in public health professionals that are prepared to develop creative and novel solutions to address challenging public health issues. These participatory approaches can be considered for use by public health agencies looking to identify and provide funding opportunities for talented early investigators, educational institutions that are preparing public health students for the workforce, or organizations that seek to provide opportunities for professional development and mentorship.

## Conclusion

Our scoping review found a wide range of studies supporting the use of crowdsourcing methods for training in public health. Future research should evaluate the impact of crowdsourcing on training outcomes.

## Supporting information

S1 Checklist Preferred Reporting Items for Systematic reviews and Meta-Analyses extension for Scoping Reviews (PRISMA-ScR) checklist. (DOCX)

S1 Text Search algorithm on crowdsourcing to support public health training. (DOCX)
