# Supplementary material for: Crowdsourcing to support training for public health: A scoping review
Source: PLOS Glob Public Health. 2023 Jul 26;3(7):e0002202. doi: 10.1371/journal.pgph.0002202 (PMC10370701; doi:10.1371/journal.pgph.0002202)
Supplement: S1 Text — (DOCX) [file pgph.0002202.s002.docx]

| **PubMed – Initial search on March 14, 2022** | | |
| --- | --- | --- |
| Search # | Query | Results |
| #1 | (crowdsourcing[MESH] OR crowdsourcing[tiab] OR crowd sourced[tw] OR crowdsource[tw] OR crowd source[tw] OR crowdsourced[tw] OR crowd sourcing[tw] OR competitive behavior[tiab] OR collective intelligence[tw] OR collective wisdom[tw] OR crowd science[tw] OR citizen science[tw] OR citizen scientist[tw] OR citizen scientists[tw] OR open call[tw] OR designathon[tw] OR hackathon[tw]) | 5,311 |
| #2 | **(training[MESH] OR training support[MESH] OR mentoring[MESH] OR mentors[MESH] OR capacity building[MESH] OR training[tw] OR trainings[tw] OR trainee[tw] OR trainees[tw] OR mentor[tw] OR mentors[tw] OR mentorship[tw] OR mentorships[tw] OR mentee[tw] OR mentees[tw] OR mentoring[tw] OR development[tw] OR apprentice[tw] OR apprenticeship[tw] OR capacity building[tw])** | 4,137,606 |
| #3 | #1 AND #2 | 1,296 |

| **PubMed – Secondary search on April 5, 2023 (Filtered dates: March 14, 2022 – April 5, 2023)** | | |
| --- | --- | --- |
| Search # | Query | Results |
| #3 | #1 AND #2 | 221 |

| **CINAHL – Initial search on March 14, 2022** | | |
| --- | --- | --- |
| Search # | Query | Results |
| #1 | TI(“crowdsourcing” OR “crowd sourced” OR “crowdsource” OR “crowd source” OR “crowdsourced” OR “crowd sourcing” OR “competitive behavior” OR “collective intelligence” OR “collective wisdom” OR “crowd science” OR “citizen science” OR “citizen scientist” OR “citizen scientists” OR “open call” OR “designathon” OR “hackathon”)  OR  AB(“crowdsourcing” OR “crowd sourced” OR “crowdsource” OR “crowd source” OR “crowdsourced” OR “crowd sourcing” OR “competitive behavior” OR “collective intelligence” OR “collective wisdom” OR “crowd science” OR “citizen science” OR “citizen scientist” OR “citizen scientists” OR “open call” OR “designathon” OR “hackathon”)  OR  MH(“crowdsourcing”) | 1,549 |
| #2 | **TI(“training” OR “mentoring” OR “capacity building” OR “trainings” OR “trainee” OR “trainees” OR “mentor” OR “mentors” OR “mentorship” OR “mentorships” OR “mentee” OR “mentees” OR “development” OR “apprentice” OR “apprenticeship”)**  OR  **AB(“training” OR “mentoring” OR “capacity building” OR “trainings” OR “trainee” OR “trainees” OR “mentor” OR “mentors” OR “mentorship” OR “mentorships” OR “mentee” OR “mentees” OR “development” OR “apprentice” OR “apprenticeship”)** | 630,003 |
| #3 | #1 AND #2 | 236 |

| **CINAHL – Secondary search on April 5, 2023 (Filtered dates: March 2022 – April 2023)** | | |
| --- | --- | --- |
| Search # | Query | Results |
| #3 | #1 AND #2 | 28 |

| **Cochrane Library – Initial search on March 14, 2022** | | |
| --- | --- | --- |
| Search # | Query | Results |
| #1 | crowdsourcing OR "crowd sourced" OR crowdsource OR "crowd source" or crowdsourced OR "competitive behavior" OR "collective intelligence" OR "collective wisdom" OR "crowd science" OR "citizen science" OR "citizen scientist" OR "citizen scientists" OR “open call” OR “designathon” or “hackathon”: ti,ab,kw (Word variations have been searched) | 521 |
| #2 | training OR trainings OR trainee OR trainees OR mentor OR mentors OR mentorship OR mentorships OR mentee OR mentees OR mentoring OR development OR apprentice OR apprenticeship): ti,ab,kw (Word variations have been searched) | 310,287 |
| #3 | #1 AND #2 | 256 |

| **Cochrane Library – Secondary search on April 5, 2023 (Filtered dates: March 14, 2022 – April 5, 2023)** | | |
| --- | --- | --- |
| Search # | Query | Results |
| #3 | #1 AND #2 | 56 |

| **Embase – Initial search on March 14, 2022** | | |
| --- | --- | --- |
| Search # | Query | Results |
| #1 | 'crowdsourcing'/exp OR 'crowdsourcing':ti,ab,kw OR 'crowd sourced':ti,ab,kw OR 'crowdsource':ti,ab,kw OR 'crowd source':ti,ab,kw OR 'crowdsourced':ti,ab,kw OR 'crowd sourcing':ti,ab,kw OR 'competitive behavior':ti,ab,kw OR 'collective intelligence':ti,ab,kw OR 'collective wisdom':ti,ab,kw OR 'crowd science':ti,ab,kw OR 'citizen science':ti,ab,kw OR 'citizen scientist':ti,ab,kw OR 'citizen scientists':ti,ab,kw OR 'open call':ti,ab,kw OR 'designathon':ti,ab,kw OR 'hackathon':ti,ab,kw | 5,427 |
| #2 | 'training'/exp OR 'training support'/exp OR 'mentoring'/exp OR 'mentors'/exp OR 'capacity building'/exp OR 'training':ti,ab,kw OR 'trainings':ti,ab,kw OR 'trainee':ti,ab,kw OR 'trainees':ti,ab,kw OR 'mentor':ti,ab,kw OR 'mentors':ti,ab,kw OR 'mentorship':ti,ab,kw OR 'mentorships':ti,ab,kw OR 'mentee':ti,ab,kw OR 'mentees':ti,ab,kw OR 'mentoring':ti,ab,kw OR 'development':ti,ab,kw OR 'apprentice':ti,ab,kw OR 'apprenticeship':ti,ab,kw OR 'capacity building':ti,ab,kw | 4,996,697 |
| #3 | #1 AND #2 | 1,524 |

| **Embase – Secondary search on April 5, 2023 (Filtered publication years: 2022, 2023)** | | |
| --- | --- | --- |
| Search # | Query | Results |
| #3 | #1 AND #2 | 327 |

| **Global Health – Initial search on March 14, 2022** | | |
| --- | --- | --- |
| Search # | Query | Results |
| #1 | TI(“crowdsourcing” OR “crowd sourced” OR “crowdsource” OR “crowd source” OR “crowdsourced” OR “crowd sourcing” OR “competitive behavior” OR “collective intelligence” OR “collective wisdom” OR “crowd science” OR “citizen science” OR “citizen scientist” OR “citizen scientists” OR “open call” OR “designathon” OR “hackathon”)  OR  AB(“crowdsourcing” OR “crowd sourced” OR “crowdsource” OR “crowd source” OR “crowdsourced” OR “crowd sourcing” OR “competitive behavior” OR “collective intelligence” OR “collective wisdom” OR “crowd science” OR “citizen science” OR “citizen scientist” OR “citizen scientists” OR “open call” OR “designathon” OR “hackathon”) | 592 |
| #2 | **TI(“training” OR “mentoring” OR “capacity building” OR “trainings” OR “trainee” OR “trainees” OR “mentor” OR “mentors” OR “mentorship” OR “mentorships” OR “mentee” OR “mentees” OR “development” OR “apprentice” OR “apprenticeship”)**  OR  **AB(“training” OR “mentoring” OR “capacity building” OR “trainings” OR “trainee” OR “trainees” OR “mentor” OR “mentors” OR “mentorship” OR “mentorships” OR “mentee” OR “mentees” OR “development” OR “apprentice” OR “apprenticeship”)** | 427,401 |
| #3 | #1 AND #2 | 126 |

| **Global Health – Secondary search on April 5, 2023 (Filtered dates: March 14, 2022 – April 5, 2023)** | | |
| --- | --- | --- |
| Search # | Query | Results |
| #3 | #1 AND #2 | 1 |

| **Total number of records identified from the initial and secondary searches** | | |
| --- | --- | --- |
| Search date | Query | Results |
| March 14, 2022 | Initial search | 3,438 |
| April 5, 2023 | Secondary search | 633 |
|  | Total | 4,071 |
