# Supplementary material for: Crowdsourcing to support training for public health: A scoping review
Source: PLOS Glob Public Health. 2023 Jul 26;3(7):e0002202. doi: 10.1371/journal.pgph.0002202 (PMC10370701; doi:10.1371/journal.pgph.0002202)
Supplement: Attachment — Submitted filename: PGPH-D-22-01976 - Response to Reviewers.docx [file pgph.0002202.s003.docx]

Dear *PLoS Global Public Health* Editor,

Thank you for these helpful comments that we have used to strengthen the paper. Please find below our response to reviewers (point-by-point responses). We have also submitted a clean version and a tracked changes version of the manuscript.

Thank you for your consideration.

Sincerely,

Kadija Tahlil and Joseph Tucker on behalf of the authors

--------------------------

Editor Comments:

**COMMENT 1.** “In the online submission form, you indicated that "The data used and analyzed during this study are available from the corresponding author upon reasonable request.". All PLOS journals now require all data underlying the findings described in their manuscript to be freely available to other researchers, either 1. In a public repository, 2. Within the manuscript itself, or 3. Uploaded as supplementary information.
This policy applies to all data except where public deposition would breach compliance with the protocol approved by your research ethics board. If your data cannot be made publicly available for ethical or legal reasons (e.g., public availability would compromise patient privacy), please explain your reasons by return email and your exemption request will be escalated to the editor for approval. Your exemption request will be handled independently and will not hold up the peer review process, but will need to be resolved should your manuscript be accepted for publication. One of the Editorial team will then be in touch if there are any issues.”

**RESPONSE 1.** All data underlying our findings are described within the manuscript.

**COMMENT 2.** “Please add a full list of legends for all your Supporting Information files after the references list.”

**RESPONSE 2.** We have now added a full list of legends for our Supporting Information files after the references list. This revision can be found on page 33, lines 746 - 749 of the tracked changes version of the manuscript.

**COMMENT 3.** “Some material included in your submission may be copyrighted. According to PLOS’s copyright policy, authors who use figures or other material (e.g., graphics, clipart, maps) from another author or copyright holder must demonstrate or obtain permission to publish this material under the Creative Commons Attribution 4.0 International (CC BY 4.0) License used by PLOS journals. Please closely review the details of PLOS’s copyright requirements here: PLOS Licenses and Copyright. If you need to request permissions from a copyright holder, you may use PLOS’s Copyright Content Permission form.

Please respond directly to this email or email the journal office and provide any known details concerning your material’s license terms and permissions required for reuse, even if you have not yet obtained copyright permissions or are unsure of your material’s copyright compatibility.

Potential Copyright Issues:

Fig 2: Please confirm whether you drew the images / clip-art within the figure panels by hand. If you did not draw the images, please provide (a) a link to the source of the images or icons and their license / terms of use; or (b) written permission from the copyright holder to publish the images or icons under our CC-BY 4.0 license. Alternatively, you may replace the images with open source alternatives. See these open source resources you may use to replace images / clip-art:
- <https://commons.wikimedia.org>
- <https://openclipart.org/>”

**RESPONSE 3.** We did not draw the images within Fig 2. The source of our images was Canva.com. The images we used from Canva are free to use. According to Canva’s content license agreement, one of the permitted uses of Canva designs can be in online or electronic publications (<https://www.canva.com/policies/content-license-agreement/>).

Reviewer 1 Comments:

**COMMENT 1.** “The manuscript lacks a registered study protocol as recommended by the latest scoping review guidelines (Tricco et al., 2018).”

**RESPONSE 1.** We have now registered our scoping review protocol with the Open Science Framework. We have added the following sentence in the Methods section: “We registered the protocol for the scoping review in the Open Science Framework (Registration DOI: <https://doi>.org/10.17605/OSF.IO/Q3PNH).” This revision can be found on page 5, lines 123 - 125 of the tracked changes version of the manuscript.

**COMMENT 2.** “Could the authors make the introduction punchier? Why is the crowdsourcing approach crucial? Is it more economical? Does it have a greater engagement capacity than other approaches? Does it have greater capillarity? Why is this an important topic for the field of global health? Would it support especially low- and middle-income countries? Would this type of approach help with fast responses to public health emergencies? In which way?”

**RESPONSE 2.** We agree the introduction can be more punchier. We have added the following language in the Introduction section: “Traditional training programs often involve experts delivering one-way instruction and guidance to trainees with the aim of enhancing their personal and professional development. Crowdsourcing can be used to shift from conventional training approaches to a more open and collaborative process. Instead of experts being primarily responsible for training methods and outcomes, a diverse group of individuals from the community can work together to frame training strategies. Crowdsourcing to support training can help to prepare public health practitioners for interdisciplinary partnerships and provide access to community-developed resources.” This revision can be found on page 4, lines 98 – 105 of the tracked changes version of the manuscript.

**COMMENT 3.** “The research question is not clearly described.”

**RESPONSE 3.** We have now clarified our research question and purpose for the scoping review. We have included the following language at the end of the Introduction section: “Hence, this scoping review investigates and summarizes the extent to which crowdsourcing has been used to support and promote public health training and explores critical components of how crowdsourcing can be used to improve public health training.” This revision can be found on pages 5, lines 115 – 118 of the tracked changes version of the manuscript.

**COMMENT 4.** “There are updated guidelines on how to conduct scoping reviews. Please see in: doi: 10.11124/JBIES-21-00064; doi: 10.1186/s13012-022-01223-6; doi: 10.7326/M18-0850. The authors chose a framework from 2003 [Arksey and O’Malley, 2003]. The authors should justify why they chose this approach instead of the more updated ones or describe their efforts to guarantee the manuscript is adjusted to reflect the latest and updated recommendations.”

**RESPONSE 4.** In addition to the Arksey and O’Malley framework, we followed the PRISMA Extension for Scoping Reviews (doi: 10.7326/M18-0850). We updated the following sentence in the Methods section: “We organized a scoping review of the literature, drawing on the framework of Arksey and O’Malley [14] and following the PRISMA Extension for Scoping Reviews (S1 File).” This revision can be found on pages 5, lines 122 – 123 of the tracked changes version of the manuscript. We also submitted a completed PRISMA checklist as supporting information (S1 File).

**COMMENT 5.** “The authors should clarify how search terms were identified. Are they all “medical subject headings,” merely keywords, or both?”

**RESPONSE 5.** We agree a clarification is needed on how search terms were identified. We included the following language in the Methods section: “We identified and adapted these search terms from prior crowdsourcing and training review literature.” This revision can be found on page 5, lines 132 – 133 of the tracked changes version of the manuscript. Also, the search strategy included both medical subject headings and keywords. The updated search algorithm is provided as supporting information (S2 File).

**COMMENT 6.** “The authors should describe narrative synthesis in detail, mainly: (1) Which information was extracted from the publications? What methodology do authors apply to gather results? (2) What categories/domains emerged from the reading? (3) Who extracted the content (only one author)? Two authors? (4) If disagreements surge about including or not a text fragment, how did the author solve it?”

**RESPONSE 6.** We have provided more detail about our narrative synthesis and data analysis process. We included the following language in the Methods section: “Two reviewers independently reviewed titles and abstracts for inclusion, and a third reviewer was available to resolve discrepancies. Then two independent reviewers examined full-text manuscripts, excluding studies based on the criteria above. Data were extracted about the purpose of crowdsourcing, crowdsourcing method (open call, designathon, other), country income level, type of crowdsourcing event (digital, in-person or both), whether open access materials were generated, and topic of the crowdsourcing event. We categorized crowdsourcing based on the Joint International Consensus Statement on Crowdsourcing [17]. Subsequently, a narrative synthesis of the extracted data was performed. The stages of our narrative synthesis included: 1) descriptive statistics to summarize the extent and nature of included studies and 2) thematic categorization, which involved identifying common training areas and grouping studies based on those training categories.” This revision can be found on pages 6 – 7, lines 152 – 162 of the tracked changes version of the manuscript.

**COMMENT 7. “**The discussion section did not adequately contextualize the findings and discuss related research.”

**RESPONSE 7.** We have provided more detail and context to our discussion section. These revisions can be found on pages 22 – 25, lines 307 – 378 of the tracked changes version of the manuscript.

Reviewer 2 Comments:

**COMMENT 1.** “Line 52: search terms related to crowdsourcing and training alone? Public health?”

**RESPONSE 1.** We did not include terms related to public health in our search strategy because we only searched medical and public health research databases for our scoping review. Also, one of our inclusion criterion was that articles had to be pertinent to public health. Therefore, we evaluated each article for its public health relevance. We have updated the following language in the Abstract: “We searched five medical and public health research databases using terms related and training.” This revision can be found on page 2, line 52 of the tracked changes version of the manuscript.

**COMMENT 2**. “Line 52: Search done on March 14^th^, 2022, given it’s over a year now, consider repeating search to pick up any new articles relevant to the review.”

**RESPONSE 2.**  We agree that an updated search is important to capture any new and relevant articles. We have conducted a secondary search on April 5^th^, 2023 and included an additional 14 studies to our scoping review. We have revised our manuscript to reflect this updated search.

**COMMENT 3**. “Line 53: reword the sentence to read ---For this review, the concept of crowdsourcing included open calls, designathons, and other activities. For the others is there a definite list of what other activities were considered as crowdsourcing for this SR?”

**RESPONSE 3.** We have revised the sentence to now read: “For this review, the concept of crowdsourcing included open calls, designathons, and other activities.” This revision can be found on page 2, line 53 of the tracked changes version of the manuscript.

We did not create a definite list of other crowdsourcing activities. However, we categorized an activity as “other” if the crowdsourcing method was not an open call or a hackathon. The specific crowdsourcing activity for each included article can be found in Tables 2-6.

**COMMENT 4.** “Lines 71-82: the opening paragraph relies heavily on the WHO TDR open call on mentorship. This is good and sets the scene but would be great to also emphasize crowdsourcing as being an innovative approach to support training and some of the benefits this offers.”

**RESPONSE 4.** We have added the following language in the Introduction section: “Traditional training programs often involve experts delivering one-way instruction and guidance to trainees with the aim of enhancing their personal and professional development. Crowdsourcing can be used to shift from conventional training approaches to a more open and collaborative process. Instead of experts being primarily responsible for training methods and outcomes, a diverse group of individuals from the community can work together to frame training strategies. Crowdsourcing to support training can help to prepare public health practitioners for interdisciplinary partnerships and provide access to community-developed resources.” This revision can be found on page 4, lines 98 – 105 of the tracked changes version of the manuscript.

**COMMENT 5**. “Line 85: typo here at the beginning of the paragraph- change “the” to “there””

**RESPONSE 5.** Thank you for catching this typo. We have fixed the following sentence in the Introduction: “There is an increased recognition that we need to provide inclusive training to support diverse trainees [5].” This revision can be found on page 3, line 87 of the tracked changes version of the manuscript.

**COMMENT 6.** “Line 88-89: crowdsourcing has been used to identify LMIC researchers for training in crowdfunding and public engagement in health research as part of a WHO/TDR global programme”

**RESPONSE 6.** We have revised the following language in the Introduction section: “Crowdsourcing has been previously used to identify LMIC researchers for training opportunities and engagement in health research as part of the WHO/TDR global programme [8,9].” This revision can be found on pages 3 - 4, lines 90 – 92 of the tracked changes version of the manuscript.

**COMMENT 7.** “Line 113: consider updating the search to pick up any new articles relevant to the review”

**RESPONSE 7.** We agree that an updated search is important to capture any new and relevant articles. We have conducted a secondary search on April 5^th^, 2023 and included an additional 13 studies to our scoping review. We have revised our manuscript to reflect this updated search.

**COMMENT 8.** “Line 141-142: Please add a sentence detailing how the extracted was analysed into review findings and presented (add references as relevant)”

**RESPONSE 8.** We included the following language in the Methods section: “The stages of our narrative synthesis included: 1) descriptive statistics to summarize the extent and nature of included studies and 2) thematic categorization, which involved identifying common training areas and grouping studies based on those training categories.” This revision can be found on pages 6 – 7, lines 159 – 162 of the tracked changes version of the manuscript.

**COMMENT 9.** “Was the review registered as a protocol in an open access repository?”

**RESPONSE 9.** We have now registered our scoping review protocol with the Open Science Framework. We have added the following sentence in the Methods section: “We registered the protocol for the scoping review in the Open Science Framework (Registration DOI: <https://doi>.org/10.17605/OSF.IO/Q3PNH).” This revision can be found on page 5, lines 123 - 125 of the tracked changes version of the manuscript.

**COMMENT 10.** “Table 1: first- The section on purpose says 13 studies developed learning materials and the next session says 14 studies generated open access materials? Secondly, I am not sure of the variable “open access materials generated” in table 1 since not all papers included had the purpose of generating materials.”

**RESPONSE 10.** Thank you for this comment. We have updated Table 1 to include the relevant articles found during our secondary search. Not all studies that used crowdsourcing to develop learning materials made their resources available to the general public or without any restriction. You are correct that not all studies had the purpose of generating open access materials, but we wanted to highlight studies that did produce open access resources. An important component to crowdsourcing is sharing solutions, ideas or findings to the public, and one method of sharing is providing open access materials. We elaborate on this aspect of crowdsourcing in the Discussion section: “Crowdsourcing comes with an obligation to give back to the public who created the idea. Therefore, learning materials that were developed from crowdsourcing can then be used for future education and capacity-building. In the 13 studies that used crowdsourcing to develop learning materials, eight studies made their materials widely available to the public [11, 40, 41, 43, 48, 49, 52, 53], which allows the resources to be accessible without restrictions.

” This revision can be found on pages 22 – 23, lines 327 – 332 of the tracked changes version of the manuscript.

**COMMENT 11.** “Line 303: 13 studies used crowdsourcing to develop learning materials, eight developed open access materials, this might be confusing for the reader going with the results presented in table 1 that suggests 14 included articles developed open access materials. Please review the presentation of these results.”

**RESPONSE 11.** We have revised the sentence to now read: “In the 13 studies that used crowdsourcing to develop learning materials, eight studies made their materials widely available to the public [11, 40, 41, 43, 48, 49, 52, 53], which allows the resources to be accessible without restrictions.” This revision can be found on pages 22 – 23, lines 327 – 332 of the tracked changes version of the manuscript.

**COMMENT 12.** “Line 380-383: Refs 8 and 9 appear to be incomplete with title of the articles missing”

**RESPONSE 12.**  Thank you for catching this error. We have updated refs 8 and 9 to as follows:

8. Ulitin A, Mier-Alpaño JD, Labarda M, Juban N, Mier AR, Tucker JD, et al. Youth social innovation during the COVID-19 pandemic in the Philippines: a quantitative and qualitative descriptive analyses from a crowdsourcing open call and online hackathon. *BMJ Innov.* 2022;8:161–168.
9. Tan RKJ, Mpofu R, Kay P, Sebastian D, Novakovic D, Lo YJ, et al. Audio innovation and songs to spur change in global health: evidence from a national crowdsourcing open call for youth social innovation in Malaysia. *BMJ Innov.* 2022;8:199–206.

These revisions can be found on page 26, lines 412 – 419 of the tracked changes version of the manuscript.
