# Supplementary material for: Crowdsourcing to support training for public health: A scoping review
Source: PLOS Glob Public Health. 2023 Jul 26;3(7):e0002202. doi: 10.1371/journal.pgph.0002202 (PMC10370701; doi:10.1371/journal.pgph.0002202)
Supplement: Attachment — Submitted filename: PGPH-D-22-01976R1 - Response to Reviewers.docx [file pgph.0002202.s004.docx]

Dear PLOS Global Public Health Editor,

Thank you for these helpful comments that we have used to strengthen the paper. Please find below our response to reviewer #1 (point-by-point responses). We have also submitted a clean version and a tracked changes version of the manuscript.

Thank you for your consideration.

Sincerely,

Kadija Tahlil and Joseph Tucker on behalf of the authors

--------------------------

Reviewer 1 Comments:

**COMMENT 1. “**Introduction (lines 80-82): The definition of training should be moved from the introduction to the methods section.”

**RESPONSE 1.** We have now moved the definition of training from the Introduction to the Methods section. We have added the following sentence in the Methods section: “For this paper, we define training broadly to encompass formal education, informal education, mentorship, coaching, and capacity-building for a wide range of ages and learning backgrounds [16].” This revision can be found on pages 5-6, lines 136 – 138 of the tracked changes version of the manuscript.

**COMMENT 2.** “2.1. Please, review the PRISMA-ScR Checklist. The authors answered 'no' to the following statement: "Indicate whether a review protocol exists; state whether and where it can be accessed (e.g., via a Web address); and, if available, provide registration information, including the registration number."”

**RESPONSE 2.** We have reviewed the PRISMA-ScR Checklist. Please see the updated S1 File. The following sentence regarding whether a review protocol exists and its registration number can be found in the Methods section: “We registered the protocol for the scoping review in the Open Science Framework (Registration DOI: <https://doi.org/10.17605/OSF.IO/Q3PNH)>.” This sentence can be found on page 5, lines 120 – 122 of the tracked changes version of the manuscript.

**COMMENT 3.** “2.2. S2 File and PRISMA FLOWCHART. Please ensure the reader can follow the final results considered. The results available in S2 File need to match the results in the box 'Records identified from the initial search´. I am wondering if the authors considered the results #3; #1, and #2 for each database. If so the final count (n = 4.071 citations) does not match the number of 3.438 citations available in 'Records identified from the initial search. Could the authors clarify, please?”

**RESPONSE 3.** We agree that a clarification is required to help readers follow the screening and selection process. Please see the updated S2 File, which includes an additional table that clarifies the number of records retrieved from the initial and secondary searches and the total number of records (page 6). We also updated the caption of the PRISMA flow diagram with the following language: “The total number of records identified from the initial (n = 3,438) and secondary (n = 633) searches is 4,071. The secondary search was performed after records from the initial search were assessed at full text review.” This revision can be found on pages 7, lines 178 – 180 of the tracked changes version of the manuscript.

**COMMENT 4.** “Results: It would be helpful if the authors could clarify how your results address the paper's two main questions. The narrative categories 1) educating participants, 2) developing learning materials, 3) promoting mentorship, and 4) identifying trainees appear to address the first question. However, I could not find the results for question two [(2) What are the Critical components of How crowdsourcing can be used to improve public health training?] described in detail. It would be relevant if the authors provided a more thorough explanation in this regard.”

**RESPONSE 4.** We have now clarified what we mean by critical components of how crowdsourcing can be used to enhance public health training. These components include the specific method of crowdsourcing, the setting (country income level) in which crowdsourcing is hosted, the type of interaction between individuals participating and organizing the crowdsourcing event, and whether open access materials are generated for future use. We have revised the following sentence in the Methods section: “Data were extracted about the purpose of crowdsourcing and health topic of the crowdsourcing event. We also extracted information on the following components of crowdsourcing that can support public health training: crowdsourcing method (open call, designathon, other), country income level, type of crowdsourcing event (digital, in-person or both), and whether open access materials were generated.” We believe this clarification will provide the context needed to understand the Results and Discussion sections. This revision can be found on pages 6, lines 153 – 158 of the tracked changes version of the manuscript.
